# Effects of a Behavior-Based Weight Management Program Delivered Through a State Cooperative Extension and Local Public Health Department Network, North Carolina, 2008-2009

**Published:** 2011-06-15

**Authors:** Lauren MacKenzie Whetstone, Kathryn M. Kolasa, Carolyn Dunn, K. S. U. Jayaratne, Surabhi Aggarwal, Sherée Vodicka, Lori Schneider, Cathy Thomas, Meg van Staveren, Carolyn Lackey

**Affiliations:** East Carolina University, Greenville, North Carolina; East Carolina University Brody School of Medicine; North Carolina Cooperative Extension, North Carolina State University, Raleigh, North Carolina; North Carolina Cooperative Extension, North Carolina State University, Raleigh, North Carolina; North Carolina Cooperative Extension, North Carolina State University, Raleigh, North Carolina; North Carolina Division of Public Health, Raleigh, North Carolina; North Carolina Division of Public Health, Raleigh, North Carolina; North Carolina Division of Public Health, Raleigh, North Carolina; North Carolina Division of Public Health, Raleigh, North Carolina; North Carolina State University, Raleigh, North Carolina

## Abstract

**Introduction:**

Eat Smart, Move More, Weigh Less (ESMMWL) is an adult weight management program developed in response to North Carolina Obesity Plan recommendations to make weight management interventions accessible to underserved populations. ESMMWL was designed to be delivered through the North Carolina Cooperative Extension and North Carolina Division of Public Health. Program coursework included content on evidence-based eating and physical activity behaviors and incorporated mindful eating concepts. The objectives of this study were to describe participant changes in weight and behaviors and to document the effectiveness of the program.

**Methods:**

In this prospective pilot study, courses were delivered and data collected from January 2008 through June 2009. Instructors provided feedback about implementation. For participants, height, weight, and waist circumference were measured at baseline and completion. Participants completed a questionnaire about changes in their eating and physical activity behaviors, changes in their confidence to engage in weight management behaviors, and their satisfaction with the course.

**Results:**

Seventy-nine instructors delivered 101 ESMMWL courses in 48 North Carolina counties. Most of the 1,162 completers were white women. Approximately 83% reported moving toward or attaining their goal. The average weight loss was 8.4 lb. Approximately 92% reported an increase in confidence to eat healthfully, and 82% reported an increase in confidence to be physically active. Instructors made suggestions for program standardization.

**Conclusion:**

This study demonstrated the effectiveness, diffusion, and implementation of a theoretically based weight management program through a state extension and local public health department network. Study of the sustainability of changes in eating and physical activity behaviors is needed.

## Introduction

Eat Smart, Move More North Carolina is a statewide obesity prevention campaign launched in 2001 and administered by the North Carolina Division of Public Health. The campaign engages more than 60 organizations. In 2006, a team representing several state organizations developed an obesity plan (1). The plan recommends making weight management interventions accessible to underserved populations among the state's more than 2 million overweight or obese adult residents.

In response to this plan, a multi-agency team recommended the development and delivery of a weight management program through existing infrastructures of the North Carolina Cooperative Extension and North Carolina Division of Public Health. Instructors would be county cooperative extension agents and health department health promotion coordinators (HPCs). As with most extension programs, instructors would have flexibility in delivering an Eat Smart, Move More, Weigh Less (ESMMWL) course to meet their community needs. Both agencies had experience delivering such programs; extension agents requested an update to their 25-year-old curriculum.

A curriculum writing team determined that the ESMMWL course would offer classes focused on the 12 evidence-based eating and physical activity behaviors for weight management (2), use the theory of planned behavior (3), and incorporate acceptance strategies such as "living mindfully" (4-6). The curriculum was peer reviewed by state and local nutrition and physical activity professionals and a family physician. Nineteen lessons focused on known predictors of successful weight management such as eating more fruits and vegetables and being physically active (2). The curriculum includes methods for planning and tracking these behaviors (7). Mindful eating concepts such as acknowledging personal responses to food without judgment and being aware of and reflecting on the effects of eating in response to emotional or environmental issues (6) were included in each lesson. Potential instructors were trained for delivery of ESMMWL. During training, instructors studied factors associated with successful weight management programs, such as use of incentives (2,8). Extension agents and HPCs decided on the number and sequence of lessons, course fee, inclusion of activity breaks during the lessons, food demonstrations, additional handouts, and types of incentives. Details of ESMMWL, including references, PowerPoint presentations, marketing materials, a participant magazine, a food and physical activity diary, a participant evaluation questionnaire, and instructor summary form and training materials, are published elsewhere (9).

The objective of this study was to describe changes in weight, body mass index (BMI), and waist circumference, as well as mindfulness and confidence in ability to follow eating and physical activity behaviors that contribute to weight management among participants who completed an ESMMWL course. This study also documents the performance of ESMMWL.

## Methods

### Study design and evaluation measures

This pilot program included training instructors, implementing ESMMWL in North Carolina counties, and measuring participant changes in weight, waist circumference, health behaviors, and confidence in ability to engage in physical activity and healthful eating. Instructors delivered courses in their own county between January 2008 and June 2009. Start and end dates varied by instructor. The North Carolina State University institutional review board approved the study for the protection of human participants.

### Participant outcomes

At the first meeting, participants recorded their sex, race or ethnicity, age, and goal of weight maintenance or loss. Participants were encouraged to set a goal of losing no more than 2 lb per week. Pairs of participants, guided by instructors, measured their height and beginning and ending waist circumference and weight. BMI was calculated by 1 author (K.J.). Participants who attended the last class and completed measurements and an evaluation questionnaire, provided by the instructor, were considered "completers." The 30-item questionnaire documented self-reported changes in mindfulness and the 12 eating and physical activity behaviors taught in ESMMWL. It was developed by the writing team and reviewed by an evaluation specialist (9). Using a 5-point Likert scale (very low to very high), participants rated their confidence in engaging in the behavior both before and after the program, and they reported whether changes were a result of program participation. Participants reported their past participation in weight management classes and satisfaction with the ESMMWL course and described their weight as a "lifelong struggle" or a new concern. Data were entered and analyzed by 2 authors (K.J., L.W.). Two summary confidence scores were calculated as measures of program effectiveness. The score for confidence in their ability to engage in physical activity was based on being physically active more or less than 30 minutes per day and participating in strength training, and possible scores ranged from 3 to 15. The score for confidence in their ability to follow healthful eating behaviors was based on responses for 8 distinct healthful eating practices, and possible scores ranged from 8 to 40. Change scores (before and after program) were calculated for BMI, weight, waist circumference, and the 2 confidence scores for comparisons by race and sex. Change in the confidence scores was compared for weight-loss status and history of weight struggle and course participation.

### Program performance

The variables used to describe program performance included the number of instructors who delivered a course compared with the number trained; the number, frequency, and length of classes taught, course location, fees charged, and incentives provided; and participants' satisfaction and instructor comments. A course was "delivered" if instructors returned participant data and the instructor summary form that documented the number, frequency, duration, and site of the classes, fee charged, use of incentives, number of participants enrolled and completed, and instructor feedback. In a follow-up telephone survey of all trained instructors conducted by an author (C.D.), plans for future course offerings were documented. Participant dropout rate was calculated on the basis of the number of participants who completed the measurements and questionnaire on the last class day compared with the number with initial measurements only. The course was made available to adults who wanted "to lose weight, maintain a healthy weight, or learn healthier lifestyle behaviors" (9,10). Fees for the course were established by the instructor, based on the instructor's perception of what the market could bear and, in some cases, factoring in a partial rebate-type incentive for participants who completed all or most of the classes. Instructors used various avenues to market the program, including local newspapers, e-mail to existing clients, or flyers at worksites. Participant characteristics were used to determine whether specific audiences (eg, African Americans vs whites, past participants in weight-loss program vs first-timers) met their goals, were more mindful, or experienced changed confidence in eating or physical activity behaviors.

### Statistical analysis

Data were analyzed using SPSS version 16 (SPSS, Inc, Chicago, Illinois). Frequency distributions and descriptive statistics were used to summarize participants' responses. Independent-samples *t* tests and Pearson correlations were used to describe the bivariate relationships between BMI changes and participant characteristics and ESMMWL course characteristics to determine what audience might complete a weight management course using this approach. We conducted a multiple linear regression with change in BMI as the outcome variable and participant and course characteristics as independent variables.

## Results

### Participant outcomes

Of the 1,162 participants who completed ESMMWL, most were white women. The mean age was 51.8 years. Participants identified an average weight-loss goal of 15.5 lb (range, 0-76 lb); 83% reported that they moved toward or attained their goal. Most completers (87%) lost weight; the average was 8.4 lb (range, 0.1-44 lb). The means for BMI, weight, waist circumference, confidence in ability to be physically active, and confidence in ability to eat healthfully improved significantly after participation (Table 1). Approximately 92% of participants reported an increase in confidence in their ability to eat healthfully, and 82% reported an increase in confidence in their ability to be physically active. Changes in BMI, weight, and waist circumference were significantly different by race (Table 2). Participants who gained weight had significantly smaller changes in confidence scores for physical activity and for healthful eating, on average, than those who lost weight (Table 3).

At the conclusion of their course, most participants reported changes in eating behaviors, physical activity, and mindfulness (Table 4). They also reported increased confidence in their ability to engage in these healthy behaviors.

### Program performance

Seventy-nine instructors (53 extension agents, 26 HPCs) delivered 101 ESMMWL courses in 48 counties between January 2008 and June 2009. An additional 26 instructors planned to teach in the near future. Fifty ESMMWL courses were delivered at worksites and the rest in community settings such as faith organizations. Instructors taught an average of 16 lessons (range, 8-19) in 15 weeks (range, 8-24); most met weekly (97%) for 1 hour (83%). An average of 24 participants enrolled in a course, and 54% completed it. Participants paid fees ranging from $5 to $150. Approximately 80% paid $25 or less. Instructors reported that they could charge a fee for ESMMWL that would be less expensive than a clinical consult for weight management or fees being charged by commercial weight-loss programs, worksites, or hospital wellness facilities in their communities. Incentive(s) including giveaways, money, and time off from work were offered in 55% of the courses.

Variables that were significantly related to change in BMI (incentives, cost, number of lessons attended, confidence to eat healthfully and be physically active, and weight-loss goal) were included in a linear regression model to identify independent predictors (Table 5). Larger improvements in BMI were associated with greater change in confidence in ability to eat healthfully, a larger weight-loss goal, a greater number of weeks participating, and higher cost of the program.

Approximately 60% of participants reported a lifelong struggle with their weight; this was the first weight-loss class for approximately 33% of participants. More African Americans than whites reported that this was their first course (*χ* ^2^ = 14.22, *P* < .001). Preprogram BMI was highest among those who had struggled with their weight and been in courses before. Change in confidence in ability to eat healthfully was significant and was greatest among participants who had struggled with their weight most of their lives and who were taking their first course (Table 6).

Approximately 97% of completing participants said that ESMMWL met their expectations, and 99% said they would recommend the program to others. Most participants were satisfied or very satisfied with the quality of instructors' presentations, instructors' knowledge, program materials, and overall quality of the program.

Instructors responded positively to the ESMMWL magazine and PowerPoint presentations, noting they contained major concepts in a concise, easily understood, colorful, and visually appealing presentation. Some said handouts were needed in addition to the magazine. Instructors reported that it was difficult to engage participants for 19 weeks and the course could be shortened by deleting repetition and combining some topics. They found it difficult to motivate participants to complete the food and physical activity diary for the entire course. They stated that attendance varied because of work conflicts and personal issues.

## Discussion

This evidence-based weight management program included strategies to promote mindful eating and physical activity to a large number of participants through the existing infrastructure of the extension and local health departments. Participants in the ESMMWL course experienced significant and positive changes that contribute to weight management. Most participants who completed a course lost weight at a rate consistent with standards for weight control programs for low-risk patients in North Carolina (11), reduced their waist circumference, and increased their confidence in their ability to eat healthfully and be physically active. Their results were comparable to results reported in self-help, worksite, and nonclinical commercial programs (12-16). Changes in participants' BMI and weight were associated with increased confidence in the ability to eat healthfully and be physically active. Almost all reported being more mindful about eating and physical activity behavior. These results add to the literature that a mindful approach can be effective for weight management, at least in the short term.

No recent published reports of comparable noncommercial weight management programs were identified for comparison of program performance. The state agencies found that ESMMWL had an acceptable reach; a large number of agents and HPCs incorporated ESMMWL into their work plan. Reaching consumers in all counties, however, may require increasing the pool of trained instructors to include dietitians, exercise physiologists, and health educators. We report an average dropout rate of 46%, which is higher than the rates reported in studies of commercial weight-loss and clinical programs (12). Instructors commented that participants dropped out for the same reasons as cited in other studies (13), including lack of time, program did not suit them, personal issues, and health limitations. The expected attrition rate for a noncommercial weight management program delivered at the community level is unknown. ESMMWL performed acceptably both for people seeking their first organized effort at weight loss and those who have been through other programs. Some outcomes differed by race/ethnicity. Further exploration is needed to understand how to reach more men and whether some course content should be tailored by race/ethnicity. On the basis of data from this study, along with instructor feedback and participant evaluation comments, ESMMWL has been standardized as a 15-lesson course, and online training for instructors, based on the live training, has been developed (17).

Our study had some limitations. This weight management program was developed on the basis of published reports but was conducted in real-world settings and without a control group. The behavior changes and changes in confidence were based on self-report by course completers. At the time of this pilot, a validated measure of mindful eating was not available (18). We did not have the resources to characterize participants who dropped out nor to determine whether the outcomes were sustained after participation in ESMMWL ended.

This study demonstrated the effectiveness, wide diffusion, and implementation of a theoretically based weight management program that included concepts of mindfulness through a state extension and local health agency network. Among participants who completed an ESMMWL course, changes in weight, BMI, and waist circumference were significant, as were changes in confidence in ability to eat healthfully and be physically active. Further study of the sustainability of these changes is needed.

## Figures and Tables

**Table 1:** Characteristics of Participants Before and After Completion of Eat Smart, Move More, Weigh Less, North Carolina, 2008-2009

**Characteristic**	Precourse Mean (SD)	Postcourse Mean (SD)	n	*t* a
Body mass index, kg/m^2^	32.7 (8.0)	31.5 (7.8)	1,085	31.50
Weight, lb	193.7 (45.4)	186.8 (44.1)	1,133	32.12
Waist circumference, in	40.2 (10.4)	38.5 (10.2)	807	23.92
Overall confidence for doing physical activity^b^	7.1 (3.0)	10.3 (2.6)	981	38.18
Overall confidence for eating healthfully^c^	23.7 (6.4)	32.4 (4.6)	941	43.88

Abbreviation: SD, standard deviation.

a All differences are significant at *P* < .001.

b Range is from 3 to 15.

c Range is from 8 to 40.

**Table 2:** Changes in Characteristics of Participants, by Race, After Completion of Eat Smart, Move More, Weigh Less, North Carolina, 2008-2009

**Characteristic**	Race/Ethnicity	n	Change in Characteristic, Mean (SD)	*F*	*P* Value
Body mass index, kg/m^2^	African American	212	−0.86 (1.05)	10.83	<.001
	White	755	−1.29 (1.27)		
	Other	36	−0.92 (1.10)		
Weight, lb	African American	226	−5.16 (6.11)	10.93	<.001
	White	787	−7.60 (7.56)		
	Other	36	−5.38 (6.23)		
Waist circumference, in	African American	159	−1.31 (1.93)	3.83	.02
	White	546	−1.77 (2.02)		
	Other	30	−2.08 (1.83)		
Overall confidence in ability to be physically active^a^	African American	189	3.01 (2.61)	0.90	.41
	White	702	3.22 (2.55)		
	Other	33	3.58 (2.51)		
Overall confidence in ability to eat healthfully^b^	African American	180	9.46 (6.43)	1.87	.15
	White	676	8.58 (5.82)		
	Other	30	7.87 (6.44)		

Abbreviation: SD, standard deviation.

a Range is from 0 (no change) to 12 (maximum change).

b Range is from 0 (no change) to 32 (maximum change).

**Table 3:** Changes in Confidence Indicators, by Weight Change Category, Among Participants Who Completed Eat Smart, Move More, Weigh Less (N = 1,162), North Carolina, 2008-2009

**Indicator**	Change in Confidence in Ability to be Physically Active, Mean (SD)^a^	Change in Confidence in Ability to Eat Healthfully, Mean (SD)^b^
Gained weight	2.38 (2.68)	6.31 (5.05)
Stayed the same weight	2.25 (2.27)	5.79 (4.47)
Lost weight	3.32 (2.58)	9.03 (6.10)

Abbreviation: SD, standard deviation.

a Range is from 0 (no change) to 12 (maximum change). *F*_2,956_ = 7.91, *P* < .001.

b Range is from 0 (no change) to 32 (maximum change). *F*_2,918_ = 11.37, *P* < .001.

**Table 4:** Changes in Behavior Indicators Among Participants Who Completed Eat Smart, Move More, Weigh Less (N = 1,162), North Carolina, 2008-2009

**Indicator**	Participants Who Attributed Behavior to Program, %	Participants Who Were Already Engaging in Behavior, %
Am more mindful of what and how much I eat	91.8	7.1
Eat smaller portions	88.6	6.5
Eat fewer calories	87.6	6.0
Am more mindful of getting physical activity each day	85.6	11.1
Eat less fast food	76.4	19.0
Eat 2-3 cups of vegetables most days	73.4	15.8
Eat 1½-2 cups of fruit most days	73.3	13.7
Prepare and eat more meals at home	66.2	28.4
Am physically active at least 30 min/d	57.4	22.6
Drink fewer calorie-containing beverages	56.8	40.4
Eat breakfast most days	51.5	43.4
Include strength training	50.5	10.8
Am physically active more than 30 min/d	40.7	12.7

**Table 5:** Independent Participant and Course Predictors of Change in Body Mass Index, Eat Smart, Move More, Weigh Less, North Carolina, 2008-2009

**Predictor**	β	*t*	*P* Value
Change in confidence in ability to eat healthfully	.173	3.10	.002
Change in confidence in ability to be physically active	.105	1.90	.06
Weight-loss or maintenance goal	.292	6.61	<.001
Program incentive offered	−.087	−1.96	.05
Cost of program	.088	1.96	.02
Weeks participated	.920	4.24	<.001

**Table 6:** Confidence in Ability to Change, by Experience With Prior Weight-Loss Programs, Among Participants Who Completed Eat Smart, Move More, Weigh Less, North Carolina, 2008-2009

Characteristic	Change in Confidence in Ability to be Physically Active, Mean (SD)^a^	Change in Confidence in Ability to Eat Healthfully, Mean (SD)^b^
Struggled with weight most of life. First time in a weight-loss program.	3.46 (2.88)	10.30 (6.27)
Struggled with weight most of life. Attended other programs in past.	3.22 (2.55)	8.42 (5.92)
Have not struggled with weight. First time in a weight-loss program.	2.90 (2.40)	8.40 (5.99)
Have not struggled with weight. Attended other programs in past.	3.12 (2.61)	7.72 (5.89)

Abbreviation: SD, standard deviation.

a Range is from 0 (no change) to 12 (maximum change). *F*_3,964_ = 1.66, *P* = .18

b Range is from 0 (no change) to 32 (maximum change). *F*_3,924_ = 6.07, *P* < .001.
